# Giant Basal Cell Carcinoma in a Renal Transplant Recipient: A Case Report

**DOI:** 10.7759/cureus.58956

**Published:** 2024-04-24

**Authors:** Nikolaos Garmpis, Dimitrios Dimitroulis, Anna Garmpi, Paraskevi Ioanna Tasioula, Christos Damaskos

**Affiliations:** 1 NS Christeas Laboratory of Experimental Surgery and Surgical Research, Medical School, National and Kapodistrian University of Athens, Athens, GRC; 2 Department of Surgery, Sotiria General Hospital, Athens, GRC; 3 Second Department of Propedeutic Surgery, Laiko General Hospital, Medical School, National and Kapodistrian University of Athens, Athens, GRC; 4 First Department of Propedeutic Internal Medicine, Laiko General Hospital, Medical School, National and Kapodistrian University of Athens, Athens, GRC; 5 Renal Transplantation Unit, Laiko General Hospital, Athens, GRC

**Keywords:** skin cancer, immunosuppresion, depression, renal transplantation, basal cell carcinoma

## Abstract

Basal cell carcinoma is a skin cancer with a more benign clinical course, compared to other skin cancers. However, when left neglected, it can cause serious morbidity and mortality. A basal cell carcinoma larger than 5 cm is defined as giant. Common causes of these carcinomas are negligence, immunosuppression, low socioeconomic status, physical or mental dysfunction, light exposure, exposure to radiation, existence of a previous lesion, recurrence after treatment, and aggressive histologic pattern. In some cases, giant basal cell carcinoma has been described to infiltrate multiple intracranial structures and to be associated with distant metastasis. Herein, we present a case of a giant basal cell carcinoma on the temporary scalp of a renal transplant recipient with depression.

## Introduction

Basal cell carcinoma (BCC) constitutes a non-melanocytic skin cancer, which derives from basal cells [1]. It is usually located on the face, mostly on the nose, and less frequently on the trunk and extremities [2]. It is characterized by slow growth, local infiltration, and low metastatic potential. Neglected tumors can lead to local invasion, bone destruction, and severe morbidity. A BCC is defined as a giant when its size exceeds 5 cm [3,4].

Giant BCC constitutes less than 0.5% of all BCC lesions [5], but is related to impaired quality of life due to its size and its tendency for local invasion and elevated risk of metastasis [6]. Giant BCCs usually develop due to patient negligence and loss of follow-up [6].

Herein, we present a rare case of a giant BCC on the scalp of an elderly renal transplant recipient. This case report demonstrates a giant BCC which was developed due to patient negligigence in the context of comorbid depression.

## Case presentation

**Figure 1:**
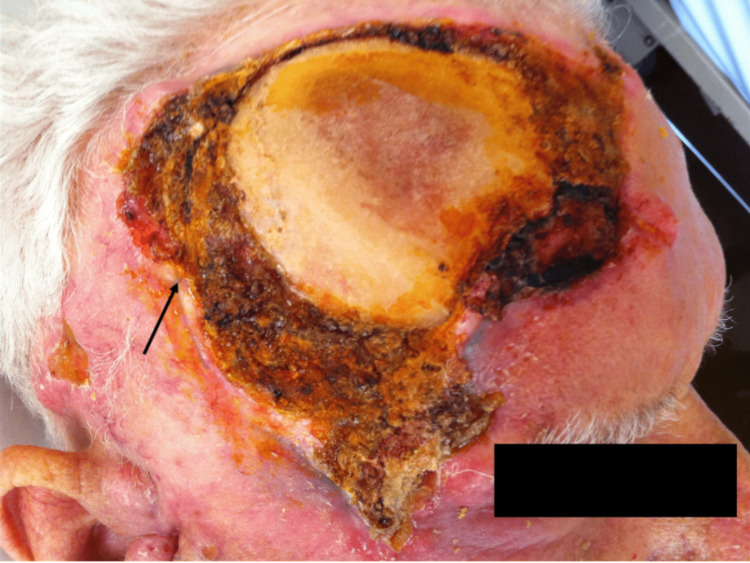
Giant basal cell carcinoma in an 85-year-old renal transplant recipient. Giant basal cell carcinoma occupies the largest part of the right temporal scalp.

A 85-year-old renal transplant recipent under immunosuppressive therapy, presented with a significant sore, occupying the largest part of his right temporal scalp (Figure 1). He also complained about headache and general malaise. His medical history included diabetes mellitus type 2, ex-smoker, depression, and arterial hypertension. He was a farmer.

Upon presentation, the vital signs were all normal. The lesion was giant, and hemorrhage, necrosis, and bone infiltration were noticed. The lesion was a lobulated, red-yellow ulcerated mass with leaking fluid and dimensions 9 x 5.5 x 4 cm. The extent of the disease was quite progressed due to the patient’s negligence and delay in seeking medical advice. The patient said that the lesion was slowly growing over the last year. No similar lesions were noticed in the body of the patient.

**Figure 2:**
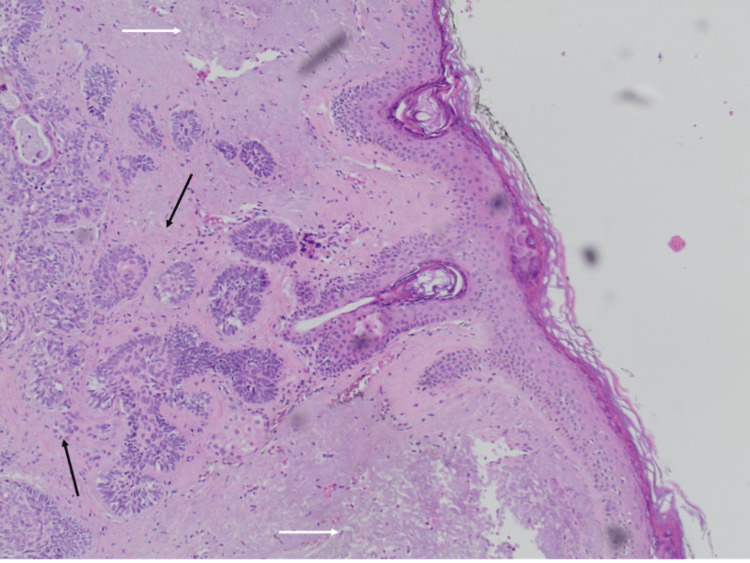
Histopathological findings. Micronodular basal cell carcinoma (hematoxylin and eosin staining, x10). There is a microfollicular growth pattern with moderate peripheral palisading. Artifact retraction is absent (black arrows). The carcinoma is located mainly in the papillary dermis with partial extension to the reticular dermis. Accompanying alterations of solar elastosis are presented (white arrows).

A computed tomography (CT) of the head/neck/chest and abdomen showed no metastatic lesions. The patient underwent partial surgical excision of the affected skin. The histopathological examination demonstrated columnar cells with keratin spots and lymphocyte infiltration. All these were consistent with BCC (Figure 2).

The patient received radiotherapy afterward. Although the patient initially showed signs of improvement locally with skin slowly growing back to cover the defect, his general condition slowly deteriorated, and he passed from other medical conditions before his scalp had grown back.

## Discussion

Giant BCCs are relatively rare. Various cases have been reported in the literature with different locations of the lesions [7,8]. This rare subtype of BCC has a more aggressive biological behavior and in some cases, it invades and infiltrates extra-dermal tissue and is associated with distant metastasis [9]. Cipullo et al. described a rare case of a giant BCC in the scalp that infiltrated multiple intracranial structures [10]. Kwon et al. reported recurrent giant BCC of the scalp with invasion of dura mater in seven patients, while Naumann et al. described a case of a recurrent giant BCC with an aggressive growth from the forehead through the skull and into the frontal lobe [11,12].

The most common causes of these BCCs are negligence, immunosuppression, exposure to light and/or radiation, the existence of a previous lesion, recurrence after treatment, and aggressive histologic pattern [7]. They are also associated with those with low socioeconomic status, and physical or mental dysfunction or disorder which influences access to health care [13]. Bartos et al. presented a case of a woman suffering from a giant ulcerating BCC of the head, infiltrating the skull and penetrating into the cranial cavity, causing compression of the brain, due to negligence, as the patient refused medical examination [14]. Hudson et al. presented a case of giant BCC developed in an autistic patient. This tumor’s growth was attributed to the mental illness of the patient [6].

Our patient was a farmer and had undergone renal transplantation. He was also suffering from depression. Thus, he had various risk factors for developing a giant BCC. These tumors have higher metastatic potential and recurrence rates, and the lungs as the most common site of metastasis [15]. Our patient showed no metastases, at the time of diagnosis.

Wide surgical excision is the cornerstone treatment in giant BCCs. Acceptable surgical margins are between 5 mm to 1 cm [9]. Vismodegib, a hedgehog pathway inhibitor (HPI) is the first US Food and Drug Administration (FDA)-approved drug for advanced BCC that is inoperable or recurred after surgery [16]. This agent is used as monotherapy or after surgical excision, with response rates of 43% for locally invasive BCC [5]. Vismodegib has been used for BCC debulking prior to surgery with favorable outcomes [17].

Chemotherapy can be also administered for metastatic, inoperable, or locally advanced BCC [18]. Cisplatin-based chemotherapy is the most commonly used chemotherapy [19]. Few reports have described the successful treatment of giant BCC with topical imiquimod 5% cream [20].

Our patient was not suitable for an aggressive surgery, and both his age and general status did not permit us to proceed with vismodegib or chemotherapy administration. Radiation was used as a palliative treatment for the pain.

## Conclusions

In conclusion, this report presents a case of a giant BCC in a renal transplant recipient with depression. BCC should be suspected in patients with neglected large lesions and immunosuppression. It should also be considered in the context of a comorbid psychiatric disease.
